# The Impact of Abnormal Glucose Tolerance and Obesity on Fetal Growth

**DOI:** 10.1155/2015/847674

**Published:** 2015-04-22

**Authors:** Erin Graves, David J. Hill, Susan Evers, Kristine Van Aarsen, Brie Yama, Su Yuan, M. Karen Campbell

**Affiliations:** ^1^Department of Epidemiology and Biostatistics, Western University, London, ON, Canada N6A 5C1; ^2^Department of Physiology and Pharmacology, Western University, London, ON, Canada N6A 5C1; ^3^Lawson Health Research Institute, London, ON, Canada N6C 2R5; ^4^Department of Family Relations and Applied Nutrition, Guelph, ON, Canada N1G 2W1; ^5^Department of Obstetrics and Gynaecology, Western University, London, ON, Canada N6H 5W9; ^6^Department of Paediatrics, Western University, London, ON, Canada N6C 2R6; ^7^Children's Health Research Institute, London, ON, Canada N6C 2V5

## Abstract

*Objective*. Factors linked with insulin resistance were examined for their association with large-for-gestational-age (LGA) infant birth weight and gestational diabetes. *Study Design*. Data came from a longitudinal cohort study of 2,305 subjects without overt diabetes, analyzed using multinomial logistic and linear regression. *Results*. High maternal BMI (OR = 1.53 (1.11, 2.12)), height (1.98 (1.62, 2.42)), antidepressant use (1.71 (1.20, 2.44)), pregnancy weight-gain exceeding 40 pounds (1.79 (1.25, 2.57)), and high blood sugar (2.68, (1.53, 5.27)) were all positively associated with LGA birth. Strikingly, the difference in risk from diagnosed and treated gestational diabetes compared to women with a single abnormal glucose tolerance test (but no diagnosis of gestational diabetes) was significant (OR = 0.65, *p* = 0.12 versus OR = 2.84, *p* < 0.01). When weight/length ratio was used instead, different factors were found to be significant. BMI and pregnancy weight-gain were found to influence the development of gestational diabetes, through an additive interaction. *Conclusions*. High prepregnancy BM, height, antidepressant use, pregnancy weight-gain exceeding 40 pounds, and high blood sugar were associated with LGA birth, but not necessarily infant weight/length ratio. An additive interaction between BMI and pregnancy weight-gain influenced gestational diabetes development.

## 1. Introduction

Normal pregnancy is characterized by a progressive increase in insulin resistance, despite only minor decreases in glucose tolerance. Pregnancy alters the maternal metabolic profile away from the normal preference for glucose as an energy source and towards the use of fatty acids, conserving glucose and amino acids for the growing fetus. These metabolic alterations enable the growing fetus to develop without compromising maternal health. The impact of maternal diet on the development of insulin resistance in pregnancy is not fully understood, and has yielded mixed findings [1]. Investigation into the impact of diet on insulin resistance in type 1 or 2 diabetes has found that certain nutritional components, including Vitamin D, omega-3 fatty acids, total dietary kilocalories, and macronutrient proportions, can influence glucose homeostasis [2–5].

In the face of chronic underlying abnormalities in the maternal metabolism, which may be exacerbated by overweight or obesity, these pregnancy-related changes can result in pregnancy complications, including gestational diabetes and abnormal fetal growth. Excess fetal growth is the most common outcome of maternal insulin resistance [6]. Maternal obesity likely acts through pathways both related to and independent of insulin resistance to influence fetal growth. Both obesity and gestational diabetes, however, add individually to the risk of excess fetal growth [7–11]. Excess fetal growth is commonly measured using large-for-gestational-age (LGA), which is a birth weight at or above the 90th percentile for population standardized birth weight. LGA infants born to mothers with gestational diabetes have more fat mass than LGA infants born to nondiabetic mothers, with a direct correlation between infant fat mass and maternal fasting glucose levels [12, 13]. The risk of developing gestational diabetes is higher in obese women than in women of normal weight. After gestational diabetes develops, the level of glycemic control achieved determines the level of risk for excess fetal growth [14]. Poor glycemic control during pregnancies complicated by gestational diabetes is more likely to result in a LGA infant than those with good glycemic control [8, 12, 14, 15].

Size-for-gestational-age is a widely used categorization for fetal growth with definite advantages, including the corrections for infant sex, infant gestational age, and normal population birth weights [16]. However, the cut-points are based on statistical considerations, which results in the LGA and small-for-gestational-age (SGA) categories being composed of both constitutionally and pathologically large and small infants [17]. Recent research has shown that size-for-gestational-age does not accurately reflect the adiposity of newborns, but instead that weight/length has a stronger correlation with infant body fat [17–20]. Maternal glucose levels have been strongly linked to not just excess fetal growth, but also excess fetal adiposity [21].

Strategies which reduce the incidence of LGA birth would have implications for the health care system, the health of the mother and the health of the child. The delivery of a LGA infant requires a caesarean section more often than with smaller infants [22], which, in turn, requires a longer hospital stay and is more costly to the health care system [23]. Also, vaginally delivered LGA infants tend to experience shoulder dystocia more often than smaller infants, which also requires more medical attention [22, 24]. There are also longer-term health implications for children born with excess adiposity, including metabolic abnormalities such as obesity and overt diabetes [25–27].

In this study we examined how factors known to influence insulin resistance, such as overweight and obesity, gestational diabetes, diet and pregnancy weight gain, affect fetal growth. Initially, fetal growth was categorized according to size-for-gestational-age, with LGA status being the outcome of interest. Secondarily, multivariable analyses were repeated using the ratio of weight/length and the results compared.

## 2. Materials and Methods

Data for this study came from the Prenatal Health Project (PHP), which is a longitudinal, population based cohort of women with singleton pregnancies recruited in London, Ontario, Canada, between 2002 and 2005. This study was approved by The University of Western Ontario Review Board for Health Sciences Research Involving Human Subjects and informed consent was obtained from all participants prior to their enrolment in the study. Details of the study sample have been reported elsewhere [28]. Briefly, a population-based sample of women was recruited between 10–22 weeks gestation from ultrasound clinics in the London, Ontario area. Study participants resided in London, Ontario, and spoke English well enough to provide informed consent. Data was collected by survey on baseline sociodemographic factors, psychosocial stress, prepregnancy and early pregnancy health and nutrition, and supplemented with information extracted perinatally from maternal and newborn hospital charts. For this analysis, women from the PHP cohort were excluded if they had overt diabetes (type 1 or type 2), if they reported using Metformin [29], or if they had missing data for infant sex or birth weight.

### 2.1. Conceptualization and Measurement of Key Variables

Fetal growth was represented by size-for-gestational-age (small for gestational age (SGA), appropriate-for-gestational-age (AGA), and LGA; resp., birthweight <10th, 10th–90th, and >90th percentile for gestational age) based on published Canadian standards [16]. Newborn weight to length ratio was used as a proxy for fetal adiposity as is common in the literature.

Key independent variables included factors known to influence insulin resistance: maternal overweight or obesity (body mass index (BMI) of 25.0–29.9 and 30.0+ kg/m^2^), gestational diabetes, diet and pregnancy weight gain. BMI was calculated from self-reported prepregnancy height and weight. Gestational diabetes (a clinical diagnosis based on 2 or more abnormal glucose tolerance tests) and abnormal glucose tolerance (a single recorded abnormal test without a gestational diabetes diagnosis) were abstracted from hospital charts. Dietary factors considered included energy intake (kilocalories), percentage of total energy that came from fat, Vitamin D sufficiency and grams of omega-3 fatty acid consumed daily. Dietary variables were captured from a validated food frequency questionnaire (FFQ), which was analyzed using the CANDAT Nutrient Analysis System (Godin London, Inc., 2003) to determine participant's average daily intakes. Nutritional data were excluded from the analysis if energy intake was more than 2 standard deviations from the mean for the cohort. Vitamin D sufficiency and grams of omega-3 fatty acid consumed included both dietary consumption and supplement use. Vitamin D sufficiency was defined as meeting Health Canada's recommendations of 5 micrograms per day [30]. Average percentage of total kilocalories from fat consumed per day was calculated relative to the percentage of kilocalories from protein and carbohydrates. The percentage of kilocalories from fat was chosen as a proxy for all three proportions (fat, carbohydrates and protein), as they were interrelated and only one could be included in the model to maintain statistical independence. This choice was made on theoretical grounds and is unlikely to affect the results. Pregnancy weight gain was available as a categorical marker captured by a pregnancy risk scoring system in hospital and extracted from women's medical records. This marker was recorded on maternal charts in a risk scoring system in which women in labour were identified as having high (at or above 40 lbs) or low weight gain (at or below 20 lbs).

Potentially confounding variables included in the analysis included: maternal age, smoking status (before and during pregnancy), stress (a composite scale encompassing financial, family, psychosocial and caregiver strain [31–33]), depression (CES-D [34]), exercise during pregnancy, medication use, nausea/vomiting during pregnancy, and a history of heart disease or high blood pressure.

### 2.2. Statistical Analysis

The main data analyses were done using Statistical Analysis Software (SAS) 9.1. Initially, frequencies for each independent variable and a univariable examination of their relationship with size-for-gestational age were completed. Multivariable analyses of factors associated with fetal growth employed multinomial logistic regression to identify associations with the three levels of size-for-gestational-age, using AGA infants as the reference group, allowing for the simultaneous calculation of odds ratios for LGA and SGA. Multivariable analyses of factors associated with newborn weight/length ratio employed linear regression.

Variables with univariate *p* values of *p* ≤ 0.20 were entered into the model in blocks, in a temporal sequence based on the point in the pregnancy where they were measured to account for hypothesized mediation along the causal pathway. During the model building process, variables were retained if they achieved *p* values of *p* ≤ 0.20. For the final model, this was adjusted to *p* = 0.05. The specific variables included in each block, as well as their order, are presented in Table 1.

Three mediation pathways were hypothesized: gestational diabetes mediating the association between BMI and a LGA infant; gestational diabetes mediating the association between diet early in pregnancy and a LGA infant; and early pregnancy diet mediating the association between BMI and a LGA infant. Early pregnancy diet included four dietary variables: average kilocalories consumed per day, average percentage of kilocalories from fat per day, total grams of omega-3 fatty acid, and the sufficiency of Vitamin D consumption. The Baron and Kenny36 criteria were used to test for the presence of the hypothesized mediation.

## 3. Results

There were 3,656 women approached by recruiters for the PHP, 2,747 of which agreed to participate in the study. Of those women, 2,421 completed the prenatal survey and 2,409 had chart data available. Gestational age and birth data were abstracted for 2,383, of which 26 were duplicate participants who were recruited in two different pregnancies. For the 26, one of the pregnancies was randomly selected and the other deleted to preserve statistical independence of the study sample. Of the 2,357 women in the final PHP cohort, this analysis excluded 27 with overt diabetes, 17 without infant gender recorded, 6 without a birth weight, and 2 who used Metformin during pregnancy without having diabetes. There were 2,305 women who met the eligibility criteria for this study. Table 1 presents the frequencies for independent variables included in the analyses.

Table 2 presents the results of both univariable and multivariable logistic regression analyses for factors associated with fetal growth. None of the hypothesized mediation was found to be present in this cohort (results not shown). Factors found to be associated with LGA in the final model were prepregnancy BMI of 25.0–30.0 (OR = 1.34) or 30.0+ (OR = 1.54), height (OR = 1.94), antidepressant use (OR = 1.70), excess pregnancy weight gain (OR = 1.74), and glucose intolerance below the threshold of gestational diabetes (OR = 2.84). It should be noted that, although the association with a prepregnancy BMI of 25.0–30.0 was modest, there is evidence of a dose-response relationship between prepregnancy BMI and the odds of having a LGA infant. The odds ratios for each variable did not change substantially with intermediate steps in the model building indicating that there was no substantial confounding present. Thus only the univariable and final model results are included in Table 2.

Table 3 presents the results of multiple linear regression analyses of the factors contributing to higher infant weight/length ratio. Prepregnancy obesity (30.0+), parity, maternal height, excess pregnancy weight gain, and glucose intolerance without gestational diabetes were significantly associated with a higher infant weight/length ratio in this model. Conversely, smoking during pregnancy and insufficient weight gain were associated with significantly lower infant weight/length ratios in this model.

## 4. Discussion

### 4.1. Interpretation of Results

Prepregnancy BMI above 25.0, height, antidepressant use, excess pregnancy weight gain, and abnormal glucose intolerance are all associated with an increased odds of delivering a LGA infant. Further, the odds ratios associated with prepregnancy BMI, height, weight gain, and abnormal glucose tolerance were comparable to other studies reported in the literature [6, 8–11, 35–41].

Because taller maternal height reflects maternal early childhood nutrition, and also has a strong genetic component, one might understand maternal height not as a risk factor for the delivery of a LGA infant, but rather as a unmodifiable covariate that is associated with the delivery of a LGA infant partially due to its genetic component [42].

Treated gestational diabetes did not significantly contribute to the risk of delivering a LGA infant, but a single abnormal glucose tolerance test during pregnancy, without a clinical diagnosis of gestational diabetes, did (OR = 2.84, *p* < 0.01). The contrast in these two measures may illustrate the differences in risk between treated and untreated glucose intolerance during pregnancy. Literature on this topic is mixed but most studies seem to indicate that gestational diabetes increases the risk of delivering a LGA infant only when it is untreated [15]. Although we did not measure blood glucose control, we speculate that the lack of an association between treated gestational diabetes and LGA in this data set may suggest good glycemic control in those with diagnosed gestational diabetes. Women who were not diagnosed with gestational diabetes but did have a single documented abnormal glucose tolerance test may have had poorer glucose control, reflected in the increased odds of delivering a LGA infant. Our findings are consistent with other research which found that degree of maternal glycaemia had the highest correlation with birth size [6]. Our results show that even at blood glucose levels below what is considered clinical gestational diabetes in Canada, women are delivering a disproportionately high number of LGA infants. These findings highlight the importance of blood glucose control during pregnancy independently from a clinical diagnosis for gestational diabetes.

Taking antidepressant medication was found to significantly increase the risk of delivering a LGA infant. Recent literature has suggested that depression and obesity are often comorbid conditions characterized by increased inflammation due to the overactivity of immune cytokines [43]. Inflammation has been shown to disrupt both the hypothalamic-pituitary-adrenal (HPA) axis as well as some neuroregulatory systems, both of which play a role in the pathology of depression [44, 45]. In this way, antidepressant use may be a marker for clinical depression in our population, and its association with LGA infants may be further evidence of this environment of increased inflammation and its impact on glucose metabolism during pregnancy. We speculate that this result may be evidence of a reduction in glucose tolerance caused by the increased inflammation present due to depression, and which then manifests as poorer glycemic regulation during pregnancy and an increased risk of a LGA infant.

Total kilocalories, the percentage of kilocalories from fat, omega-3 fatty acid intake and Vitamin D consumption did not affect a woman's risk of delivering a LGA infant, possibly due to a lack of precision in these measures, which would bias the odds ratios towards the null value. Further research is needed to determine if and how diet impacts the risk of developing gestational diabetes and delivering a LGA infant. The null results could also reflect confounding by other factors with greater explanatory power and does not necessarily indicate that diet does not contribute to gestational diabetes and LGA.

We found that there was a difference in the risk factor profile of infants who were large when classified by LGA, compared to those that were large when classified using the weight/length ratio. Our results suggest not only that the factors contributing to increased infant weight/length ratio may be subtly different from those contributing to LGA status, but also that while these two measures are correlated, they are distinctly different measures. This is important given that LGA is used predominantly in the literature, sometimes for questions that may be better represented using a weight/length ratio. The differences in the risk factor profile of LGA infants, compared to infants with a high weight/length ratio, have important implications for future work in this area.

### 4.2. Strengths and Limitations

The PHP cohort is a key strength to this analysis, with its breadth of available data and the widely generalizable population upon which it is based. An additional strength is the use of a strong theoretical framework, based on biological mechanisms from the literature and epidemiological studies, which was used to guide and interpret the analysis and understand the complex relationships that exist. The use of an analytic technique that restricted the reference group to only AGA infants, instead of incorporating all “non-LGA” infants, is also a strength.

There are also some limitations to this analysis. The data used to calculate participant's BMIs were self-reported. Because prepregnancy weight was a major factor of interest in this analysis, underestimating women's weight would bias our risk estimates associated with obesity towards the null. This should be kept in mind when interpreting the results of this study, even though it is a common limitation in the literature. Additionally, weight gain was captured as categories of weight gain instead of as the actual amount of weight gained during pregnancy. As the Institute of Medicine [46] (IOM) and Health Canada [47] both recommend different amounts of weight gain during pregnancy depending on maternal BMI, our weight gain categories may misclassify overweight and obese women's weight gain. If a woman had an overweight prepregnancy BMI and gained 26 lbs during her pregnancy, she would be classified as having gained excess weight by the guidelines but would have been put into the category for appropriate weight gain in this study. This is a limitation of the data that was collected for this cohort but we believe that it would bias our results towards the null for the excess weight gain category, indicating that the effects seen here for weight gain may actually be stronger if we had been able to take the guidelines into account.

Also, our data for gestational diabetes and abnormal glucose intolerance were not collected as discrete clinical values, but instead as the presence or absence of the test results in the patient's chart. As with other studies, our nutritional data may be limited by participants reporting what they thought they should be eating instead of accurately reporting their nutritional consumption. This has been documented in other studies [48] and may help to explain our null results for the nutritional factors.

## 5. Conclusions

Prepregnancy obesity and excess pregnancy weight gain both contribute to an increased risk of delivering an LGA infant. This further supports the findings from other similar studies in the literature and points to the need for future research focusing on both individual-level and community-level strategies for managing obesity and pregnancy weight gain, as both factors are modifiable, although modification is not easily achieved.

Our study also illustrates that excess fetal growth and excess fetal adiposity may not have the same determinants; our results indicate that the same factors do not influence both outcomes in the same way.

The most striking finding of this study is the difference in LGA risk conferred by diagnosed and treated gestational diabetes (OR = 0.65, *p* = 0.12), compared to that from a single abnormal glucose tolerance test without a diagnosis of gestational diabetes (OR = 2.84, *p* < 0.01). This suggests that the odds of delivering an LGA infant are lowered by “treated” gestational diabetes [49]. Our results highlight the importance of diagnosis, but more importantly treatment as this seems to be where the real benefit exists. Future research should focus on the establishment and adoption of universal screening and diagnosis procedures for gestational diabetes. This is already underway with work such as the recommendations made by the such as the International Association of Diabetes and Pregnancy Study Groups (IADPSG) which has made recommendations for internationally recognized gestational diabetes diagnosis criteria [6, 50].

## Figures and Tables

**Table 1:** Frequency and order of variable entry in blocks.

Variables entered in block 1 (prepregnancy factors)		
Categorical		Frequency (%)

Prepregnancy BMI	<18.5	99 (4%)
	18.5–24.9	1350 (61%)
	25.0–29.9	483 (22%)
	30.0+	282 (13%)

Preexisting CVD or hypertension	Yes	72 (3%)
	No	2233 (97%)

Prepregnancy smoking	Yes	525 (23%)
	No	1767 (77%)

Parity	0	819 (36%)
	1+	1485 (64%)

Income	<$30,000	262 (12%)
	$30,000–$79,999	1096 (50%)
	$80,000	826 (38%)

Education	Did not complete high school	119 (12%)
	Completed high school only	826 (38%)
	Some education beyond high school	1096 (50%)

Continuous	*n*	Mean (±SD)

Age (years)	2305	30 (±5)

Height (cm)	2289	165 (±7)

Variables entered in block 2 (early pregnancy factors)		
Categorical		Frequency (%)

Antidepressant use	Yes	38 (2%)
	No	2267 (98%)

Depression	≥16 on CES-D (depressed)	424 (19%)
	<16 on CES-D	1864 (81%)

Smoking during pregnancy	Yes	234 (10%)
	No	2060 (90%)

Hyper emesis	Yes	389 (17%)
	No	1899 (83%)

Exercise	<3 times a week	766 (33%)
	≥3 times a week	1539 (67%)

Continuous	*n*	Mean (±SD)

Stress (standardized composite score)	2296	0 (±1)

Variables entered in block 3 (diet and nutrition)		
Categorical		Frequency (%)

Vitamin D sufficiency	Yes	1938 (84%)
	No	367 (16%)

Continuous	*n*	Mean (±SD)

Total kilocalories	2298	2028 (±746)

Percentage of total kilocalories from fat	2298	29 (±4)

Grams of omega-3 fatty acid	2294	0.16 (±0.22)

Variables entered in block 4 (late pregnancy factors)		
Categorical		Frequency (%)

Pregnancy weight gain	Insufficient	89 (4%)
	Appropriate	1932 (84%)
	Excess	284 (12%)

Gestational diabetes	None	2206 (95%)
	Abnormal glucose tolerance	26 (1%)
	Gestational diabetes	73 (3%)

Insulin use	Yes	36 (2%)
	No	2269 (89%)

**Table 2:** Determinants of size for gestational age (LGA).

	Odds ratio of LGA	
	(95% CI)	
	Univariable	Final model
Prepregnancy BMI		
<18.5	0.59 (0.33, 1.07)	0.58 (0.32, 0.94)
18.5–24.9 (ref)	—	—
25.0–30.0	1.37 (1.03, 1.81)	1.34 (1.00, 1.79)
30.0+	1.44 (1.05, 1.98)	1.53 (1.11, 2.12)

Age (per 1 year)	1.01 (0.99, 1.04)	—

Preexisting CVD or hypertension		
Yes	1.01 (0.99, 1.04)	—
No (ref)	—	

Prepregnancy smoking		
Yes	0.98 (0.84, 1.14)	—
No (ref)	—	

Parity		
0 (ref)	—	—
1+	1.11 (0.97, 1.26)	

Height (for each 10 cm change)	1.86 (1.54, 2.25)	1.98 (1.62, 2.42)

Education		
Did not complete high school	0.91 (0.59, 1.38)	—
Completed high school only	0.98 (0.70, 1.35)	
Education beyond high school (ref)	—	

Income		
<$30,000	0.83 (0.62, 1.12)	
$30,000–$80,000 (ref)	—	—
$80,000+	0.96 (0.78, 1.19)	

Stress	1.03 (0.91, 1.16)	—

Antidepressant use		
Yes	1.85 (1.32, 2.60)	1.71 (1.20, 2.44)
No (ref)	—	—

Depression		
<16 on CES-D (ref)	—	—
≥16 on CES-D	0.97 (0.82, 1.14)	

Smoking during pregnancy		
Yes	0.83 (0.65, 1.06)	—
No (ref)	—	

Hyper emesis		
Yes	0.87 (0.73, 1.03)	—
No (ref)	—	

Exercise		
≥3 times a week (ref)	—	—
<3 times a week	1.02 (0.89, 1.16)	

Total kilocalories (per 100 kcal)	1.01 (0.99, 1.02)	—

Percent kcals from fat (per 10%)	1.20 (0.89, 1.60)	—

Vitamin D sufficiency		
Yes (ref)	—	—
No	0.95 (0.81, 1.13)	

Grams of omega-3 (per 1 gram)	0.89 (0.50, 1.61)	—

Weight gain		
Insufficient	0.57 (0.32, 1.00)	0.52 (0.29, 0.94)
Appropriate (ref)	—	—
Excess	1.77 (1.25, 2.49)	1.79 (1.25, 2.57)

Gestational diabetes		
None (ref)	—	—
Abnormal glucose tolerance	2.28 (1.29, 4.02)	2.84 (1.53, 5.27)
Gestational diabetes	0.80 (0.49, 1.30)	0.65 (0.38, 1.12)

Insulin use		
Yes	1.38 (0.93, 2.06)	—
No (ref)	—	

^*^Age, preexisting CVD or hypertension, prepregnancy smoking, parity, education, income, stress, depression, smoking during pregnancy, hyper emesis, exercise, total kilocalories, percent kilocalories from fat, vitamin D sufficiency, omega-3 fatty acid consumption, and insulin use did not reach statistical significance during the model building process.

**Table 3:** Determinants of fetal adiposity using weight/length ratio (linear regression).

	Regression coefficients	
	(95% CI)	
	Univariable	Final model
Prepregnancy BMI		
<18.5	−1.72 (−3.88, 0.44)	−1.52 (−3.63, 0.60)
18.5–24.9 (ref)	—	—
25.0–30.0	0.42 (−0.64, 1.49)	0.38 (−0.67, 1.42)
30.0+	0.98 (−0.32, 2.28)	1.35 (0.07, 2.64)

Age (per 1 year)	0.13 (0.04, 0.21)	—

Preexisting CVD or hypertension		
Yes	−0.87 (−3.22, 1.47)	—
No (ref)	—	

Prepregnancy smoking		
Yes	−1.10 (−2.13, −0.06)	—
No (ref)	—	

Parity		
0 (ref)	—	—
1+	1.65 (0.76, 2.54)	1.95 (1.07, 2.83)

Height (for each 10 cm change)	2.17 (1.54, 2.79)	2.12 (1.50, 2.74)

Education		
Did not complete high school	−0.89 (−2.81, 1.03)	—
Completed high school only	−1.04 (−2.37, 0.30)	
Education beyond high school (ref)	—	

Income		
<$30,000	−0.01 (−1.41, 1.40)	—
$30,000–$80,000 (ref)	—	
$80,000+	0.27 (−0.65, 1.19)	

Stress	−0.15 (−0.58, 0.27)	—

Antidepressant use		
Yes	2.23 (−1.43, 5.88)	—
No (ref)	—	

Depression		
<16 on CES-D (ref)	—	—
≥16 on CES-D	−0.92 (−2.03, 0.19)	

Smoking during pregnancy		
Yes	−3.03 (−4.49, −1.56)	−3.01 (−4.45, −1.57)
No (ref)	—	—

Hyper emesis		
Yes	−0.82 (−1.93, 0.29)	—
No (ref)	—	

Exercise		
≥3 times a week (ref)	—	—
<3 times a week	−0.04 (−0.94, 0.86)	

Total kilocalories (per 100 kcal)	0.005 (−0.05, 0.06)	—

Percentage of kcals from fat (per 10%)	0.54 (−0.44, 1.53)	—

Vitamin D sufficiency		
Yes (ref)	—	—
No	−0.37 (−1.54, 0.80)	

Grams of omega-3 (per 1 gram)	0.52 (−1.44, 2.49)	—

Weight gain		
Insufficient	−2.16 (−4.30, −0.02)	−2.42 (−4.54, −0.30)
Appropriate (ref)	—	—
Excess	2.01 (0.74, 3.27)	2.15 (0.90, 3.40)

Gestational diabetes		
None (ref)	—	—
Abnormal glucose tolerance	4.82 (1.09, 8.54)	4.98 (1.34, 8.63)
Gestational diabetes	−0.05 (−2.53, 2.44)	0.03 (−2.42, 2.48)

Insulin use		
Yes	0.49 (−2.69, 3.95)	—
No (ref)	—	

^*^Age, preexisting CVD or hypertension, prepregnancy smoking, parity, education, income, stress, antidepressant use, depression, hyper emesis, exercise, total kilocalories, percent kilocalories from fat, vitamin D sufficiency, omega-3 fatty acid consumption, and insulin use did not reach statistical significance during the model building process.

## References

[1] Henriksen T. (2006). Nutrition and pregnancy outcome. *Nutrition Reviews*.

[2] Gunderson E. P. (2004). Gestational diabetes and nutritional recommendations. *Current Diabetes Reports*.

[3] Franz M. J., Bantle J. P., Beebe C. A. (2002). Evidence-based nutrition principles and recommendations for the treatment and prevention of diabetes and related complications. *Diabetes Care*.

[4] Steyn N. P., Mann J., Bennett P. H. (2004). Diet, nutrition and the prevention of type 2 diabetes. *Public Health Nutrition*.

[5] Merzouk H., Khan N. A. (2003). Implication of lipids in macrosomia of diabetic pregnancy: can *n*-3 polyunsaturated fatty acids exert beneficial effects?. *Clinical Science*.

[6] Metzger B. E., Lowe L. P., Dyer A. R. (2008). Hyperglycemia and adverse pregnancy outcomes. *The New England Journal of Medicine*.

[7] Rosenn B. (2008). Obesity and diabetes: a recipe for obstetric complications. *The Journal of Maternal-Fetal & Neonatal Medicine*.

[8] Alberico S., Montico M., Barresi V. (2014). The role of gestational diabetes, pre-pregnancy body mass index and gestational weight gain on the risk of newborn macrosomia: results from a prospective multicentre study. *BMC Pregnancy and Childbirth*.

[9] Kim S. Y., Sharma A. J., Sappenfield W., Wilson H. G., Salihu H. M. (2014). Association of maternal body mass index, excessive weight gain, and gestational diabetes mellitus with large-for-gestational-age births. *Obstetrics & Gynecology*.

[10] Catalano P. M., McIntyre H. D., Cruickshank J. K. (2012). The hyperglycemia and adverse pregnancy outcome study: associations of GDM and obesity with pregnancy outcomes. *Diabetes Care*.

[11] Black M. H., Sacks D. A., Xiang A. H., Lawrence J. M. (2013). The relative contribution of prepregnancy overweight and obesity, gestational weight gain, and IADPSG-defined gestational diabetes mellitus to fetal overgrowth. *Diabetes Care*.

[12] Kunz L. H., King J. C. (2007). Impact of maternal nutrition and metabolism on health of the offspring. *Seminars in Fetal and Neonatal Medicine*.

[13] Durnwald C., Huston-Presley L., Amini S., Catalano P. (2004). Evaluation of body composition of large-for-gestational-age infants of women with gestational diabetes mellitus compared with women with normal glucose tolerance levels. *American Journal of Obstetrics & Gynecology*.

[14] Hardy D. S. (1999). A multiethnic study of the predictors of macrosomia. *The Diabetes Educator*.

[15] Langer O., Yogev Y., Xenakis E. M. J., Brustman L. (2005). Overweight and obese in gestational diabetes: the impact on pregnancy outcome. *American Journal of Obstetrics & Gynecology*.

[16] Kramer M. S., Platt R. W., Wen S. W. (2001). A new and improved population-based Canadian reference for birth weight for gestational age. *Pediatrics*.

[17] Schmelzle H. R., Quang D. N., Fusch G., Fusch C. (2007). Birth weight categorization according to gestational age does not reflect percentage body fat in term and preterm newborns. *European Journal of Pediatrics*.

[18] Yau K.-I. T., Chang M.-H. (1993). Weight to length ratio—a good parameter for determining nutritional status in preterm and full-term newborns. *Acta Paediatrica*.

[19] Braga T. D. A., Lima M. D. C. (2002). Weight/length ratio: is it a good index to assess the nutritional status of full-term newborns?. *Jornal de Pediatria*.

[20] Fok T.-F., Hon K.-L., Ng P.-C. (2009). Use of anthropometric indices to reveal nutritional status: normative data from 10,226 Chinese neonates. *Neonatology*.

[21] Metzger B. E., Lowe L. P., Dyer A. R. (2009). Hyperglycemia and adverse pregnancy outcome (HAPO) study: associations with neonatal anthropometrics. *Diabetes*.

[22] Conway D. L., Langer O. (1998). Elective delivery of infants with macrosomia in diabetic women: reduced shoulder dystocia versus increased cesarean deliveries. *American Journal of Obstetrics and Gynecology*.

[23] Burns L. R., Geller S. E., Wholey D. R. (1995). The effect of physician factors on the cesarean section decision. *Medical Care*.

[24] Dildy G. A., Clark S. L. (2000). Shoulder dystocia: risk identification. *Clinical Obstetrics & Gynecology*.

[25] Wei J. N., Lin R. S., Sung F. C. (2003). Low birth weight and high birth weight infants are both at an increased risk to have type 2 diabetes among school children in Taiwan. *Diabetes Care*.

[26] Jones R. H., Ozanne S. E. (2007). Intra-uterine origins of type 2 diabetes. *Archives of Physiology and Biochemistry*.

[27] Ievins R., Roberts S. E., Goldacre M. J. (2007). Perinatal factors associated with subsequent diabetes mellitus in the child: record linkage study. *Diabetic Medicine*.

[28] Sontrop J. M., Campbell M. K., Evers S. E., Speechley K. N., Avison W. R. (2007). Fish consumption among pregnant women in London, Ontario: associations with socio-demographic and health and lifestyle factors. *Canadian Journal of Public Health*.

[29] Glueck C. J., Goldenberg N., Wang P., Loftspring M., Sherman A. (2004). Metformin during pregnancy reduces insulin, insulin resistance, insulin secretion, weight, testosterone and development of gestational diabetes: prospective longitudinal assessment of women with polycystic ovary syndrome from preconception throughout pregnancy. *Human Reproduction*.

[30] Health Canada (2006). *Dietary Reference Intakes: Reference Values for Macronutrients*.

[31] Avison W. R., Turner R. J. (1988). Stressful life events and depressive symptoms: disaggregating the effects of acute stressors and chronic strains. *Journal of Health and Social Behavior*.

[32] Wheaton B., Avison W. R., Gotlib I. H. (1994). Sampling the stress universe. *Stress and Mental Health: Contemporary Issues and Prospects for the Future*.

[33] Pearlin L. I., Mullan J. T., Semple S. J., Skaff M. M. (1990). Caregiving and the stress process: an overview of concepts and their measures. *Journal of Community Psychology*.

[34] Radloff L. S. (1977). The CES-D scale: a self-report depression scale for research in the general population. *Applied Psychological Measurement*.

[35] Ray J. G., Vermeulen M. J., Shapiro J. L., Kenshole A. B. (2001). Maternal and neonatal outcomes in pregestational and gestational diabetes mellitus, and the influence of maternal obesity and weight gain: the DEPOSIT study. *The Quarterly Journal of Medicine*.

[36] Baeten J. M., Bukusi E. A., Lambe M. (2001). Pregnancy complications and outcomes among overweight and obese nulliparous women. *American Journal of Public Health*.

[37] Cnattingius S., Bergström R., Lipworth L., Kramer M. S. (1998). Prepregnancy weight and the risk of adverse pregnancy outcomes. *The New England Journal of Medicine*.

[38] Ricart W., López J., Mozas J. (2005). Body mass index has a greater impact on pregnancy outcomes than gestational hyperglycaemia. *Diabetologia*.

[39] Ørskou J., Henriksen T. B., Kesmodel U., Secher N. J. (2003). Maternal characteristics and lifestyle factors and the risk of delivering high birth weight infants. *Obstetrics and Gynecology*.

[40] Okun N., Verma A., Mitchell B. F., Flowerdew G. (1997). Relative importance of maternal constitutional factors and glucose intolerance of pregnancy in the development of newborn macrosomia. *The Journal of Maternal-Fetal & Neonatal Medicine*.

[41] Rodrigues T., Teles T. P., Barros H. (1999). Risk factors for macrosomia in infants of nondiabetic women. *Arquivos de Medicina*.

[42] Johnston L. B., Clark A. J. L., Savage M. O. (2002). Genetic factors contributing to birth weight. *Archives of Disease in Childhood: Fetal and Neonatal Edition*.

[43] Martinac M., Pehar D., Karlovic D., Babic D., Marcinko D., Jakovljevic M. (2014). Metabolic syndrome, activity of the hypothalamic-pituitary-adrenal axis and inflammatory mediators in depressive disorder. *Acta Clinica Croatica*.

[44] Nousen E. K., Franco J. G., Sullivan E. L. (2013). Unraveling the mechanisms responsible for the comorbidity between metabolic syndrome and mental health disorders. *Neuroendocrinology*.

[45] Bornstein S. R., Schuppenies A., Wong M.-L., Licinio J. (2006). Approaching the shared biology of obesity and depression: the stress axis as the locus of gene-environment interactions. *Molecular Psychiatry*.

[46] Institute of Medicine (IOM) (2009). *Weight Gain During Pregnancy: Reexamining the Guidelines*.

[47] Health Canada (2010). *Prenatal Nutrition Guidelines for Health Professionals: Gestational Weight Gain*.

[48] Johansson G., Wikman Å., Åhrén A.-M., Hallmans G., Johansson I. (2001). Underreporting of energy intake in repeated 24-hour recalls related to gender, age, weight status, day of interview, educational level, reported food intake, smoking habits and area of living. *Public Health Nutrition*.

[49] Sermer M., Naylor C. D., Farine D. (1998). The Toronto Tri-hospital gestational diabetes project: a preliminary review. *Diabetes Care*.

[50] Metzger B. E. (2010). International Association of Diabetes and Pregnancy Study Groups recommendations on the diagnosis and classification of hyperglycemia in pregnancy. *Diabetes Care*.

